# Bringing Clarity to the Reporting of Health Equity

**DOI:** 10.1371/journal.pmed.1001334

**Published:** 2012-10-30

**Authors:** The PLOS Medicine Editors

## Abstract

The *PLOS Medicine* editors discuss the new extension to the PRISMA reporting guidelines, PRISMA for Equity (PRISMA-E), and why it is relevant to the journal and to health research more generally.

Health equity—the absence of avoidable and unfair differences in health, including access to health care—is relevant to virtually the whole of medicine and public health and encompasses much more than inequality alone. WHO [1] notes specifically that “health inequities involve more than inequality—whether in health determinants or outcomes, or in access to the resources needed to improve and maintain health—but [is also] also a failure to avoid or overcome such inequality that infringes human rights norms or is otherwise unfair.”

Inequity in health is widespread, is itself unevenly distributed globally within and between countries, and contributes to inequity in other areas of society more generally; two recent examples show that health inequity leads to disabled individuals in the UK having less access to cancer screening [2] and older people in Latin America, China, India, and Nigeria being excluded from access to health care because of health systems that finance medical services through out-of-pocket payments [3].

The WHO Commission on Social Determinants of Health (CSDH), which defined health equity as the absence of systematic differences in health, between and within countries, *that are avoidable by reasonable action* [4], makes it clear that understanding, documenting, and measuring inequity are crucial steps in determining this “reasonable action.” However, rigorous measurement and accurate evaluation of the effects of policies on health equity are not yet universal despite the CSDH calling for researchers to measure and understand the problem and assess the impact of action.

PLOS and *PLOS Medicine* have long had an interest in health equity. The first article *PLOS Medicine* ever published [5] discussed the 1994 International Conference on Population and Development (ICPD), in which unequal access to reproductive rights and its consequences were key concepts. *PLOS Neglected Tropical Diseases*' first editorial, entitled “A New Voice for the Poor” [6], noted that “The neglected tropical diseases … represent some of humankind's most ancient scourges and possibly our greatest global health disparities.” Over the years we have provided a forum for the discussion of many other aspects of health equity, including a paper published in November 2011 by Piroska Östlin and colleagues, which explicitly stated that “Influencing regional and national research priorities on equity and health and their implementation requires joint efforts towards creating a critical mass of researchers, expanding collaborations and networks, and refining norms and standards” [7].

Another specific interest of *PLOS Medicine*, that of improving the reporting and conduct of research, now aligns with the goals of health equity and this call for refining of standards noted by Östlin and colleagues. We have been involved in the development and publication of revisions to the specific guidelines CONSORT (Consolidated Standards of Reporting Trials) [8] and PRISMA (Preferred Reporting Items for Systematic Reviews and Meta-Analyses) (previously QUOROM) guidelines [9] and overarching guidelines on developing reporting guidelines [10]; we have also supported the EQUATOR initiative on guidelines more generally [11]. We are therefore particularly pleased to be publishing this month the PRISMA-Equity 2012 extension [12], drafted during a meeting earlier this year (which included one author of this Editorial, VB), which provides reporting guidelines for systematic reviews with a specific focus on health equity. Most guidelines or extensions to reporting guidelines pertain to technical aspects, for example to improve the reporting of a specific study design such as cluster randomized trials, or for a specific intervention, such as acupuncture. Instead, the PRISMA-Equity 2012 extension is specifically aimed at improving the reporting of the relatively small proportion of systematic reviews in which health equity is a key focus.

At first sight, these guidelines may not fit into the usual technical reasons for the development of reporting guidelines. One issue discussed was whether moral reasons also drove a need for these guidelines. By developing this pragmatic tool, could they do more than just bring clarity to the reporting of specific papers; for example, could a reporting guideline even change outcomes? One paper cited by Welch and colleagues shows that vitamin A [13] has the largest absolute impact on mortality reduction for children with lowest nutritional status. Having that evidence presented as clearly as possible could potentially make the difference between an intervention being appropriately targeted, or not.

One of the original reporting guidelines, CONSORT, has done an enormous amount to raise awareness of the need for good reporting in this most experimental of human studies. Has it done more? The originators of CONSORT tend to shy away from this suggestion but guideline developers sometimes say that a good guideline is like a light shone into an untidy room; it does not tidy the room but shows where the mess is. Perhaps if the light were even turned on at the beginning of a study, there would be less “mess” throughout—and published results would also be clearer and more accurate. So, by shining a light on health equity research, as Welch and colleagues suggest, by providing “structured guidance on transparently reporting these methods and results,” the PRISMA-Equity 2012 guidelines have the potential to not only improve the state of the published literature, but also to “legitimize and emphasize the importance of reporting health equity results.” We are happy to support these dual aims and will endorse the use of these guidelines.

## Abbreviations

CONSORT: Consolidated Standards of Reporting Trials
CSDH: WHO Commission on Social Determinants of Health
ICPD: International Conference on Population and Development
QUOROM: Quality of Reporting of Meta-analyses
PRISMA: Preferred Reporting Items for Systematic Reviews and Meta-Analyses
